# Paradoxes in thyroid carcinoma treatment: analysis of the SEER database 2010—2013

**DOI:** 10.18632/oncotarget.13395

**Published:** 2016-11-16

**Authors:** Ping Zhou, Shuangming Tian, Jiale Li, Yongfeng Zhao, Wengang Liu, Yan Zhang, Zheyu Hu

**Affiliations:** ^1^ Department of Ultrasound, the Third Xiangya Hospital of Central South University, China; ^2^ Information Security and Big Data Institution, Central South University, China

**Keywords:** thyroid carcinoma, epidemiology and end results (SEER) database, surgery and radiation treatment, cox regression model, propensity score

## Abstract

Thyroid cancer is a common malignant disease with high survival rate (98.1%, 2006-2012, Surveillance Epidemiology and End Results (SEER) program). In this study, we investigated the treatment paradoxes in thyroid T0 and micro-carcinoma patients. 48,234 thyroid carcinoma patients were identified from 2010 to 2013 in SEER*Stat database (version 8.2.1) released in 2016. Survival analysis showed a significantly lower thyroid carcinoma-specific survival in T0 patients compared with T1–T3 patients. In propensity score analysis, T0 patients had a similar survival curve with T1-T3 patients when lymph node and distant metastasis stages were matched. When all variables, including radiation and surgery treatment, were matched, T0 patients had significantly higher survival compared to T3 patients. These findings suggested that more metastasis and less treatment led to poorer prognosis in T0 patients. Another paradox is about thyroid micro-carcinoma. The survival rate of micro-carcinoma patients was high (4 years survival rate was 99.92%), and more than 99% micro-carcinoma patients received surgery. Interestingly, all the patients who died because of thyroid carcinoma received surgery. Survival analysis showed no difference in survival when patients stratified by surgery or radiation. In conclusion, we suggested paradoxes in thyroid carcinoma treatment: over-treated in micro-carcinoma patients and less-treated in T0 patients.

## INTRODUCTION

Thyroid carcinoma is a kind of head and neck malignant disease with increasing incidence rate [1] and high overall survival rate [2]. According to National Comprehensive Cancer Network (NCCN) guidelines, the first diagnosis step of thyroid nodules is to measure serum thyroid-stimulating hormone (TSH) level and to do ultrasound of the thyroid and central neck. Fine-needle aspiration (FNA) with biopsy examination is required for the histology confirmation.

According to American Joint Committee on Cancer (AJCC) TNM staging for thyroid cancer (7^th^ ed., 2010), thyroid tumors are staged based on the primary tumor size. T0 patients have no evidence of primary malignant tumor. But diagnosis can be confirmed by radioiodine imaging or FNA biopsy in metastasis sites. T1 tumors are less than 2 cm. T2 tumors are between 2 cm and 4 cm. T3 tumors are more than 4 cm but limited to thyroid or any tumor size with minimal extrathyroid extension. T4 tumors extend beyond the thyroid capsule to invade subcutaneous sift tissues or prevertebral fascia. T0 patients have no evidence of primary malignant thyroid tumor, but diagnosis can be made based on histological and cytological confirmation in metastasis sites.

Total lobectomy alone is recommended for patients with thyroid micro-carcinoma (T1a). Postoperative radioiodine is recommended for T1b–T4 patients. For T0 patients, no local surgery or radiation treatment was recommended. Focal papillary carcinoma (tumor size < 4 cm in diameter) with no prior radiation exposure, no lymph node metastases, and no extrathyroidal extension, lobectomy is recommended. Otherwise, complete thyroidectomy is recommended [3]. Currently, compared to unilateral lobectomy, aggressive completion thyroid resection is preferable, because it reduce the local recurrence and nodal metastasis [4]. One exception is T0 tumor. In previous study, T0 tumors have been perceived to have a low mortality rate and are featured as well-differentiated [5]. So, compared to T1–T3 patients, T0 tumors are less treated for a long term. The clinical investigations for T0 patients are rare, too.

On the other hand, T1a thyroid micro-carcinoma (tumor size less than 1 cm in diameter) is widely investigated and aggressively treated. Though lobectomy alone is recommended for thyroid micro-carcinoma [6], surgeons always decide to perform completion thyroidectomy in most clinical settings (74.3%) [7]. Recently, to eliminate severe complications and extra cost after aggressive treatment, some specialists began to oppose completion thyroidectomy and advocate unilateral lobetectomy [8–9]. Oncologists also recommend nonsurgical treatment, such as ultrasound-guided thermal laser ablation (LA), for micro-carcinoma [10–11].

The SEER program of the National Cancer Institute (NCI) is a population-based cancer registry covering approximately 30% of the population in the United States. This database is the largest publicly available and authoritative information source on cancer incidence and survival. Using this reliable and large-scale research dataset, we gathered information of 48,243 thyroid carcinoma patients diagnosed in 2010–2013. SEER database collected full information about the thyroid carcinoma T staging based on 7^th^ edition of AJCC staging system.

## RESULTS

### Demographic and clinical features

Of 48,243 patients included in this study, 47,360 cases had definite AJCC 7^th^ T stage record. 84 patients were in T0 stage, 28,067 patients were in T1 stage, 7,727 patients were in T2 stage, 9,375 patients were in T3 stage, and 2,107 were in T4 stage. The mean (SD) and median (interquartile range) age for each T stage was listed in Table 1. ROC curve analysis determined that age of diagnosis at 59 yr was the optional cutoff age that maximizes sensitivity and specificity for predicting both thyroid carcinoma-specific mortality and all-cause mortality (Supplementary Figure S1 and S2). No racial disparity was observed among T stages.

**Table 1:** Characteristics for patients with T0–T4

Covariate	level	T stages				
		T0 (*n* = 84)	T1 (*n* = 28067)	T2 (*n* = 7727)	T3 (*n* = 9375)	T4 (*n*=2107)
Age		55.12 ± 17.8457 (42.5, 66.5)	50.28 ± 14.4751 (40, 61)	47.16 ± 16.1246 (35, 58)	50.31 ± 16.5950 (38, 62)	61.33 ± 17.6463 (50,74)
Survival months		22.30 ± 14.70	21.52 ± 14.09	21.55 ± 13.94	21.50 ± 13.95	16.30 ± 14.56
All-cause mortality	No	75 (89.29%)	27632 (98.45%)	7577 (98.06%)	9131 (97.40%)	1377 (65.35%)
	Yes	9 (10.71%)	435 (1.55%)	150 (1.94%)	244 (2.60%)	730 (34.65%)
Thyroid carcinoma- specific death	No	75 (96.15%)	27632 (99.91%)	7577 (99.61%)	9131 (99.07%)	1377 (71.02%)
	Yes	3 (3.85%)	25 (0.09%)	30 (0.39%)	86 (0.93%)	562 (28.98%)
Race	white	69 (82.14%)	22981 (81.88%)	6215 (80.43%)	7379 (78.71%)	1671 (79.31%)
	black	9 (10.71%)	1872 (6.67%)	576 (7.45%)	607 (6.47%)	128 (6.07%)
	other	6 (7.14%)	3214 (11.45%)	936 (12.11%)	1389 (14.82%)	308 (14.62%)
Grade	Well-differentiated	5 (62.50%)	6122 (86.42%)	1754 (79.80%)	1894 (68.82%)	219 (20.58%)
	Moderate	2 (25.00%)	875 (12.35%)	373 (6.97%)	614 (22.31%)	116 (10.90%)
	Poorly differentiated	1 (12.50%)	87 (1.23%)	71 (3.23%)	244 (8.87%)	153 (27.52%)
	undifferentiated	0 (0.00%)	0 (0.00%)	0 (0.00%)	0 (0.00%)	576 (54.14%)
N-stage	N0	18 (22.78%)	24110 (86.17%)	6028 (78.48%)	5432 (58.19%)	721 (36.27%)
	N1	61 (77.22%)	3870 (13.83%)	1653 (21.52%)	3903 (41.81%)	1267 (63.73%)
M-stage	M0	60 (71.43%)	27992 (99.73%)	7643 (98.91%)	9137 (97.46%)	1612 (76.51%)
	M1	24 (28.57%)	75 (0.27%)	84 (1.09%)	238 (2.54%)	495 (23.49%)
Radiation	None or refused	45 (55.56%)	18232 (66.50%)	2836 (37.83%)	2751 (30.35%)	699 (34.13%)
	Radiation Beam	4 (4.94%)	108 (0.39%)	86 (1.15%)	155 (1.71%)	461 (22.51%)
	Radioisotopes	31 (38.27%)	8940 (32.61%)	4498 (60.00%)	6037 (66.61%)	845 (41.26%)
	Radioactive implants	1 (1.23%)	122 (0.44%)	70 (0.93%)	94 (1.04%)	14 (0.68%)
	Radiation beam +isotopes/implants	0 (0.00%)	16 (0.06)	7 (0.09%)	26 (0.29%)	29 (1.42%)
Surgery	Recommended but not Performed	2 (2.38%)	66 (0.24%)	34 (0.44%)	25 (0.27%)	28 (1.34%)
	Performed	55 (65.48%)	27621 (98.80%)	7513 (97.57%)	9269 (99.03%)	1644 (78.47%)
	Not recommended	27 (32.14%)	269 (0.96%)	153 (1.99%)	66 (0.71%)	423 (20.19%)

The mean (SD) survival months for each T stage were also listed in Table 1. T4 patients had significantly shorter survival months than other patients. Chi-square tests or Fisher's exact tests (*n <* 5) demonstrated that T4 patients had the highest all-cause mortality rate and thyroid carcinoma-specific mortality rate. Interestingly, both the all-cause mortality rate and thyroid carcinoma-specific mortality rate in T0 patients were significantly higher than T1–T3 patients (Table 1). Possible reasons might be that T0 patients were older than T1 to T3 patients, and had higher proportion of poorly differentiated tumor (12.5%), lymph node metastasis (77.2%) and distant metastasis (28.6%) than T1–T3 patients. T0 patients seemed to have a more progressive carcinoma than T1–T3 patients, but less T0 patients received any type of radiation or surgery (55.56% and 34.52% respectively) than T1–T3 patients (Table 1).

### Survival analysis stratified by T stages

**Figure 1:**
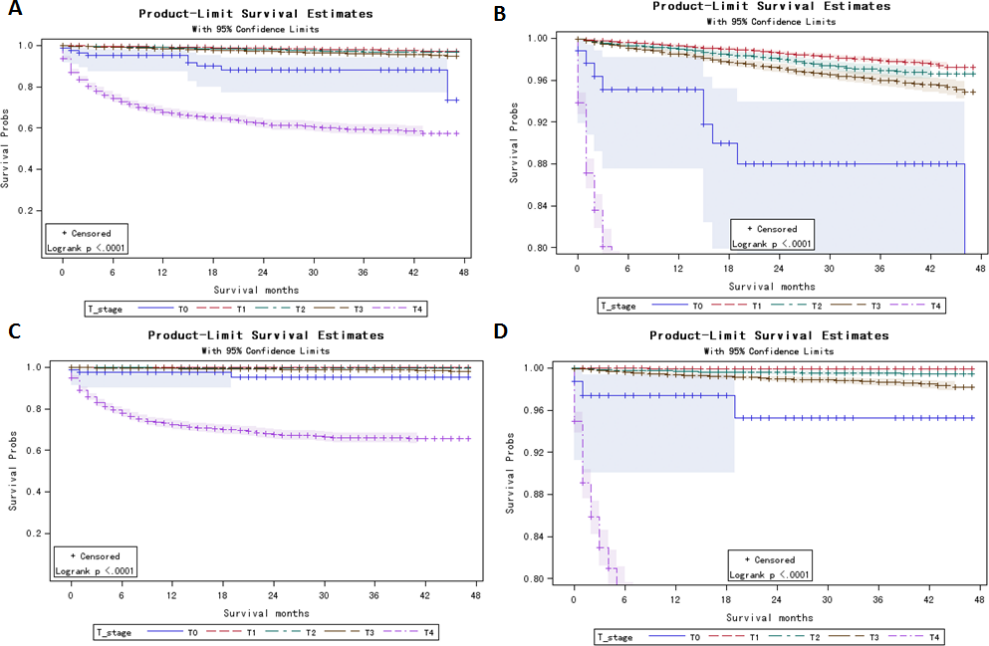
Kaplan Meier curves among patients stratified by T-stage for all cause mortality (A, B: Log rank test *p* < 0.0001) and thyroid carcinoma-specific mortality (C, D: Log rank test *p* < 0.0001)

Among 47,360 cases with definite T stage records, 1,568 all-cause deaths and 703 thyroid carcinoma-specific deaths were observed. 1-, 2- and 4-year estimated thyroid carcinoma-specific survival rates were 98.60%, 98.26% and 98.03%, respectively (Supplementary Figure S3B). Compared to T1–T3 patients, T0 patients had a poorer all-cause survival and thyroid carcinoma-specific survival. In T0 patients, the 1-, 2- and 4-year estimated survival rates were 95.12%, 88.02% and 88.02%, respectively, as compared to 99.06%, 97.14% and 95.58%, respectively, in T3 patients (Figure 1A and 1B, log-rank *P-value* < 0.001, Supplementary Table S1). In T0 patients, the 1-, 2- and 4-year thyroid carcinoma specific estimated survival rates were 97.42%, 95.25% and 95.25%, respectively, as compared to 99.38%, 98.96% and 98.48%, respectively, in T3 patients (Figure 1C and 1D, log-rank *P-value* < 0.0001, Supplementary Table S2).

### Risk factors for all-cause mortality and thyroid carcinoma-specific mortality

Univariate Cox regression analyses showed that age, black race, TNM stage, differentiation grade, radiation and surgery treatment were significant risk factors of all-cause death and thyroid carcinoma-specific death. In multivariate Cox regression model, after controlling all other influential risk factors, T0 stage did not show significant risk for all-cause death compared to T1–T3 patients (Table 2, *p >* 0.1). For thyroid carcinoma-specific death, multivariate Cox regression analysis showed that the risk of T0 patients was significantly higher than T1 patients (*p* = 0.009 by controlling other confounding factors in multivariate analysis, Table 2).

**Table 2:** Risk factors for survival: outcome is all-cause mortality and thyroid carcinoma specific mortality

Variables	level*	All cause mortality				Thyroid Carcinoma specific mortality			
		Univariate Cox regression		Multivariate Cox regression		Univariate Cox regression		Multivariate Cox regression	
		Hazard Ratio (95% CI)	*p*-value	Hazard Ratio (95% CI)	*p*-value	Hazard Ratio (95% CI)	*p*-value	Hazard Ratio (95% CI)	*p*-value
Age		1.093 (1.089, 1.097)	< 0.0001	1.054 (1.049, 1.058)	< 0.0001	1.106 (1.100, 1.112)	< 0.0001	1.042 (1.035, 1.048)	< 0.0001
Race	White	ref		ref		ref		ref	
	Black	1.287 (1.080, 1.533)	0.005	1.324 (1.091, 1.606)	0.005	1.189 (0.912, 1.550)	0.20	1.269 (0.942, 1.709)	0.12
	Other	0.952 (0.816, 1.112)	0.54	1.018 (0.859, 1.211)	0.84	1.090 (0.882, 1.347)	0.42	1.220 (0.956, 1.556)	0.11
T-stage	T0	ref		Ref		ref		ref	
	T1	0.125 (0.065, 0.243)	< 0.0001	0.754 (0.370, 1.537)	0.44	0.024 (0.007, 0.080)	< 0.0001	0.146 (0.034, 0.622)	0.009
	T2	1.177 (0.090, 0.347)	< 0.0001	1.270 (0.617, 2.613)	0.52	0.102 (0.031, 0.336)	0.0002	0.799 (0.188, 3.388)	0.76
	T3	0.244 (0.126, 0.475)	< 0.0001	1.447 (0.708, 2.955)	0.31	0.250 (0.079, 0.789)	0.02	1.534 (0.374, 6.298)	0.55
	T4	4.111 (2.130, 7.934)	< 0.0001	3.882 (1.909, 7.895)	0.0002	8.875 (2.854, 27.600)	0.0002	7.236 (1.777, 29.463)	< 0.0001
N-stage	N0	ref		Ref		ref		ref	
	N1	2.564 (2.313, 2.842)	< .0001	1.371 (1.207, 1.558)	< 0.0001	5.445 (4.654, 6.370)	<.0001	1.458 (1.220, 1.741)	< 0.0001
M-stage	M0	ref		Ref		ref	< 0.0001	Ref	
	M1	27.671 (24.902, 30.748)	< 0.0001	1.324 (1.091, 1.606)	< 0.0001	57.683 (50.043, 66.489)		2.705 (2.250, 3.252)	< 0.0001
Grade	Well differentiated	ref		Ref		ref		ref	
	Moderately differentiated	1.897 (1.335, 2.695)	0.0004	1.337 (0.934, 1.912)	0.11	2.321 (0.882, 6.106)	0.09	1.393 (0.505, 3.848)	0.52
	Poorly differentiated	24.298 (18.972, 31.118)	< 0.0001	4.865 (3.678, 6.434)	< 0.0001	149.938 (84.086, 267.362)	< 0.0001	16.443 (8.401, 32.183)	< 0.0001
	Undifferentiated	161.988 (131.507, 199.534)	< 0.0001	6.932 (5.288, 9.088)	< 0.0001	1148.482 (660.170, 1997.785)	< 0.0001	24.513 (12.598, 47.696)	< 0.0001
	Unknown	2.142 (1.761, 2.606)	< 0.0001	1.387 (1.128, 1.706)	0.002	5.657 (3.241, 9.871)	< 0.0001	3.576 (1.889, 6.772)	< 0.0001
Radiation	None or refused	ref		ref		ref		ref	
	Radiation Beam	13.924 (12.341, 15.709)	< 0.0001	0.803 (0.658, 9.943)	0.07	27.967 (24.015, 32.569)	< 0.0001	0.777 (0.643, 0.939)	0.009
	Radioisotopes	0.266 (0.230, 0.307)	< 0.0001	0.342 (0.292, 0.401)	< 0.0001	0.202 (0.156, 0.261)	< 0.0001	0.244 (0.184, 0.325)	< 0.0001
	Radioactive implants	0.473 (0.212, 1.055)	0.07	0.556 (0.248, 1.243)	0.15	0.407 (0.102, 1.635)	0.21	0.402 (0.100, 1.621)	0.20
	Radiation beam +isotopes/implants	2.775 (1.440, 5.346)	0.002	0.645 (0.331, 1.259)	0.20	6.147 (3.052, 12.378)	< 0.0001	0.805 (0.393, 1.651)	0.55
Surgery	Recommended but not Performed	ref		ref		ref		ref	
	Performed	0.060 (0.046, 0.077)	< 0.0001	0.352 (0.252, 0.491)	< 0.0001	0.040 (0.028, 0.057)	< 0.0001	0.497 (0.311, 0.795)	0.004
	Not recommended	2.021 (1.555, 2.627)	< 0.0001	1.295 (0.927, 1.811)	0.13	2.335 (1.643, 3.319)	< 0.0001	1.350 (0.851, 2.143)	0.20

### Adjusting for patient characteristics using propensity score matching

**Figure 2:**
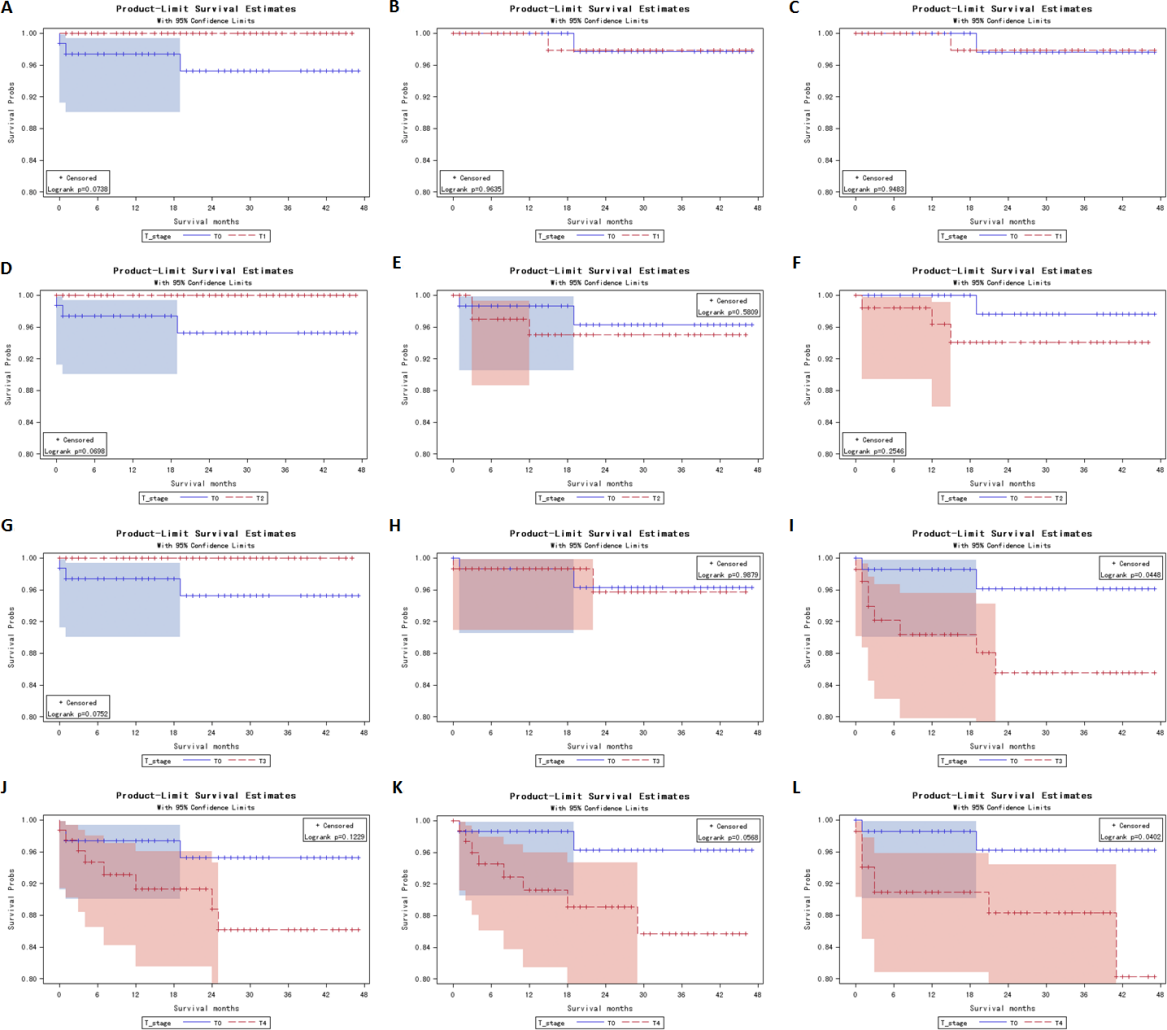
Kaplan Meier curves for matched T-stage pairs Age, race and differentiation grade matching between T0 and T1 (**A**), T0 and T2 (**D**), T0 and T3 (**G**), T0 and T4 (**J**). Age, race, differentiation grade, N / M stage matched between T0 and T1 (**B**), T0 and T2 (**E**), T0 and T3 (**H**), T0 and T4 (**K**). Age, race, differentiation grade, N / M stage, surgery and radiation treatment matched between T0 and T1 (**C**), T0 and T2 (**F**), T0 and T3 (**I**), T0 and T4 (**L**).

To account for potential bias due to an imbalance between T0 and T1–T1 patients regarding the age, race, tumor differentiation grade, N stage, M stage, surgery and radiation treatment, propensity score matching was carried out as described in Methods. In survival analysis, after propensity score matching with age, race and tumor differentiation grade, T0 stage remained a poorer prognosis for cancer-specific mortality when compared to T1, T2 and T3 stage (Log-rank test, *p* = 0.0738, 0.0698, 0.0752, respectively, Figure 2A, 2D and 2G). After propensity score matching with age, race, tumor differentiation grade, N stage and M stage, T0 patients demonstrated a similar survival curve with T0–T3 patients (Figure 2B, 2E and 2H). When all influential variables, including surgery and radiation treatment, were matched, T0 stage became a better prognosis for cancer-specific mortality, compared to T3 stage (Log-rank test, *p* = 0.0448, Figure 2I). These findings verified our hypothesis that metastasis and less treatment compared to T1–T3 patients led to poorer prognosis in T0 patients.

### Risk factors for all-cause mortality and thyroid carcinoma-specific mortality in thyroid micro-carcinoma patients

In 17,315 included micro-carcinoma patients, both univariate and multivariate Cox regression analyses showed that age, radiation beam and non-surgery were significant risk factors of all-cause death. Radioisotopes treatment was associated with better prognosis for all-cause mortality (Table 3). Multivariate Cox analysis suggested that lymph node metastasis was a risk factor of all-cause death in micro-carcinoma patients. For thyroid carcinoma-specific death, age and radiation beam treatment were demonstrated as risk factors. All 10 thyroid carcinoma-specific deaths in T1a patients received surgery. Among 9 deaths whose radiation treatment information was known, 7 did not receive any radiation therapy (Table 3).

**Table 3:** Risk factors for survival in patients with micro thyroid carcinoma patients: outcome is all cause mortality (*n* = 17,587) and thyroid carcinoma-specific mortality (*n* = 17,315)

			All cause mortality				Thyroid Carcinoma specific mortality				
Variables	level *	N^**^	Univariate Cox regression		Multivariate Cox regression		Univariate Cox regression			Multivariate Cox regression	
			Hazard Ratio (95% CI)	*p*-value	Hazard Ratio (95% CI)	*p*-value	N^**^	Hazard Ratio (95% CI)	*p*-value	Hazard Ratio (95% CI)	*p*-value
Age			1.076 (1.064, 1.087)	< 0.0001	1.074 (1.063, 1.085)	< 0.0001		1.066 (1.015, 1.119)	0.008	1.069 (1.015, 1,125)	0.01
Race	White	227/14420	ref		ref		7/14200	ref		ref	
	Black	34/1267	1.336 (0.851, 2.099)	0.21	1.447 (0.919, 2.277)	0.11	1/1234	1.652 (0.203, 13.424)	0.64	2.244 (0.264, 19.052)	0.46
	Other	21/1900	0.841 (0.525, 1.350)	0.47	1.008 (0.626, 1.622)	0.97	2/1881	2.258 (0.469, 10.868)	0.31	3.155 (0.630, 15.813)	0.16
Grade	Well differentiated	34/3759	ref		ref		2/3727	ref		ref	
	Moderately differentiated	5/432	1.309 (0.511, 3.353)	0.57	1.073 (0.418, 2.753)	0.88	0/427	0 (0, .)	0.99	0 (0, .)	1.00
	Poorly differentiated	1/35	3.119 (0.428, 22.725)	0.26	2.146 (0.292, 15.779)	0.45	0/34	0 (0, .)	0.00	0 (0, .)	1.00
	Undifferentiated	0/0	-		-		0/0	-		-	
	Unknown	242/13361	1.452 (1.002, 2.103)	0.05	1.280 (0.881, 1.860)	0.20	8/13127	1.080 (0.229, 5.086)	0.92	1.021 (0.206, 5.052)	0.98
N-stage	N0	254/15882	ref		ref		8/15636	ref		ref	
	N1	27/1651	1.323 (0.855, 1.979)	0.17	2.380 (1.524, 3.718)	0.0001	2/1626	2.397 (0.509, 11.286)	0.27	4.246 (0.638, 28.274)	0.13
M-stage	M0	281/17556	ref		ref		10/17285	ref		ref	
	M1	1/31	2.350 (0.330, 16.758)	0.39	1.680 (0.230, 12.270)	0.61	0/30	0 (0, .)	1.00	0 (0, .)	1.00
Radiation	None or refused	242/13531	ref		ref		7/13296	ref		ref	
	Radiation Beam	3/43	4.927 (1.574, 15.423)	0.006	3.713 (1.154, 11.948)	0.03	1/41	46.612 (5.734, 378.880)	0.0003	24.597 (2.114, 286.227)	0.01
	Radioisotopes	33/3598	0.600 (0.414, 0.870)	0.007	0.653 (0.434, 0.981)	0.04	1/3566	0.494 (0.061, 4.016)	0.51	0.388 (0.040, 3.770)	0.41
	Radioactive implants	1/52	1.483 (0.208, 10.583)	0.69	1.599 (0.222, 11.542)	0.64	0/51	0 (0, .)	1.00	0 (0, .)	1.00
	Radiation beam +isotopes/implants	0/5	0 (0, I)	0.98	0 (0, I)	0.97	0/5	0 (0, .)	1.00	0 (0, .)	1.00
Surgery	Recommended but not Performed	4/33	ref		ref		0/29	ref		ref	
	Performed	207/17390	0.081 (0.030, 0.217)	< 0.0001	0.119 (0.038, 0.373)	0.0003	10/17193	I (0, .)	1.00	I (0, .)	1.00
	Not recommended	10/90	1.006 (0.316, 3.209)	0.99	1.035 (0.284, 3.770)	0.96	0/80	1.00 (0,.)	1.00	0.728 (0, .)	1.00

Note: N** is demonstrated as numerator divided by denominator, where numerator indicates the number of death and denominator indicates the number of patients in each category level for every variable

### Survival analysis among thyroid micro-carcinoma patients

**Figure 3:**
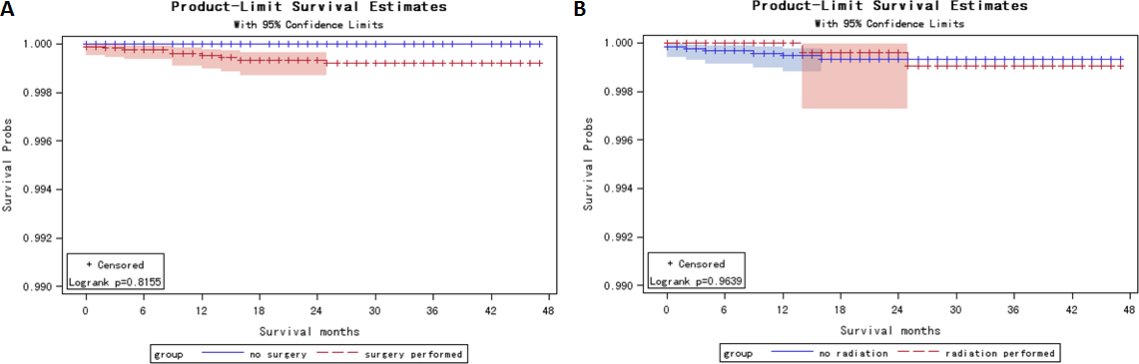
Kaplan Meier curves among thyroid micro-carcinoma patients for thyroid carcinoma specific mortality and thyroid carcinoma-specific mortality stratified by surgery treatment (A Log rank test *p* = 0.82) and radiation treatment (B Log rank test *p* = 0.96)

Unlike T0 patients (65.5% received surgery and 44.4% received radiation), 99.3% T1a patients received surgery, while only 21.46 % patients received radiation therapy. In thyroid micro-carcinoma patients, the 1-, 2- and 4-year estimated survival rate was 99.32%, 98.64% and 97.50%, respectively (Supplementary Figure S4A). The 1-, 2- and 4-year thyroid carcinoma-specific estimated survival rate was 99.95%, 99.93% and 99.92%, respectively (Supplementary Figure S4B). Kaplan Meier curves showed no significant difference in cancer-specific survival stratified by surgery (log rank *p* = 0.82) and radiation therapy (log rank *p* = 0.96) (Figure 3). The survival rate information stratified by surgery and radiation therapy was demonstrated in Supplementary Tables S3 and S4.

## DISCUSSION

Overdiagnosis and overtreatment problem are common in thyroid carcinoma [12]. On the other hand, surgeons and oncologists always ignored T0 patients due to perceived low mortality rate [5]. Our findings showed that compared to T1-T3 patients (> 97% patients underwent surgery), only 65% T0 patients received surgery. Compared to T2–T4 patients (> 60% patients underwent radiation therapy), only about 44% T0 patients received radiotherapy (Table 1).

Based on SEER data 2011–2013, T0 stage had a significantly poorer thyroid carcinoma-specific survival and all-cause survival compared to T1–T3 patients (Figure 1). More metastasis and less treatment compared to T1–T3 patients were the two major reasons. For example, compared to T3 patients (41.81% lymph node metastasis and 2.54% distant metastasis), 77.22% T0 patients had lymph node metastasis and 28.57% had distant metastasis. But, only 65.48% and 44.44% T0 patients received surgery and radiation therapy, significantly lower than T3 patients (99.03% received surgery and 69.65% received radiation) (Table 1).

Propensity score matching analysis further verified our hypothesis. As shown in Figure 2, T0 patients still showed marginally lower survival rate when age, race and histology grade were matched with T1–T3 patients (Log-rank test, *p* = 0.0738, 0.0698, 0.0752, respectively, Figure 2A, 2D and 2G). When N stage and M stage were matched, T0 patients showed similar survival curve with T1–T3 patients. This finding suggests that late-stage in terms of nodes or metastases lead to poorer prognosis in T0 patients. Moreover, when radiation and surgery treatment were matched, T0 patients had significantly higher survival compared to T3 patients. This means that if T0 patients had the same metastasis stage and treatment with T3 patients, T0 patients would have a better prognosis.

As for the possible mis-staged problem in T0 patients, the chance is small. SEER database records actual primary tumor size information in column “CS tumor size 2004+”, and records T stage information in column “Derived AJCC T (7^th^ ed, 2010)”. Since T stages were defined based on primary thyroid tumor sizes, the patients' T stages were consistent to the corresponding actual primary tumor sizes in this study.

Among thyroid micro-carcinoma patients, even the 4-year survival rate was 99.92% (Supplementary Figure S4), still more than 99% patients received surgery, and 21.55% patients received both surgery and radiation therapies. Thyroid carcinoma-specific survival analysis suggested that no survival difference was observed in micro-carcinoma patients stratified by surgery or radiation therapy (Figure 3, Supplementary Table S3 and S4). Multivariate Cox regression analysis showed that, by controlling other factors, radiation beam therapy was a risk factor of all-cause mortality with a hazard ratio of 3.713 (95% CI: [1.154, 11.948], Table 3). Radioisotopes and surgery strategies cause irreparable life-long damages to human organs and lead to several morbidities, including hypothyroidism, salivary gland disturbance and vocal disorders [13–19]. Recommendation for nonsurgical treatment strategy includes ultrasound-guided thermal laser ablation (LA). It is safe, repeatable and works well for tumors less than 15 mm [20–21]. This nonsurgical and repeatable strategy would be promising in future.

## MATERIALS AND METHODS

### Database

The SEER*Stat database, which was released by the Surveillance Research Program at the National Cancer Institute (NCI) in 2016, was used as the data source in the present study [22]. 48,234 patients diagnosed as thyroid carcinoma (ICCC site recode ICD-O-3/WHO 2008 and Behavior code ICD-O-3: malignant) were identified in the SEER 18 Research Data + Hurricane Katrina Impacted Louisiana Cases, Nov 2015 Sub (1973–2013 varying) incidence database. Because SEER database records detailed T stage (AJCC 2010) information from 2010, so we only included cases diagnosed from 2010 to 2013.

### AJCC T staging

To compare the survival rate among different T stages, 47,360 patients were categorized according to AJCC T staging (2010). SEER database records the actual tumor size. T stages were defined based on primary thyroid tumor size. In this study, the T stages were consistent to the corresponding actual tumor size.

Demographics, lymph node metastasis, distant metastasis, differentiation grade, surgery treatment (surgery performed, recommended but not performed, and not recommended) and radiation treatment (radioisotopes, beam radiation, radioactive implants, combination of beam with implants or isotopes, and none) were evaluated in patients with different T categories. To investigate the treatment efficiency in thyroid micro-carcinoma (T1a) patients, 17,315 T1a patients were categorized based on surgery and radiation treatment types. Survival analysis was performed to evaluate the treatment efficiency.

### Statistical analysis

Patients were followed up until December 2013. The primary outcome measures thyroid carcinoma-specific mortality. The secondary outcome measures the all-cause mortality. The candidate risk factors included age, race, differentiation grade, surgery type, radiation type, and TNM stage. Numeric variables were summarized as the mean (standard deviation) or median (interquartile range), where appropriate. Categorical variables were reported as counts (percentage). An analysis of variance was used to compare continuous variables with symmetric distributions among different treatment types and T staging categories. Chi-square tests or Fisher's exact tests (*n <* 5) were used to compare categorical variables among T staging categories or treatment group. The Kaplan-Meier method was used to plot the survival distributions, and the log-rank test was used to assess differences in survival experience between the groups. The Cox proportional hazards regression was performed to estimate the hazard ratio to identify the risk factors for thyroid carcinoma-specific mortality and all-cause mortality. To further adjust for potential baseline confounders, a propensity score matching was carried out. A receiver operating characteristic (ROC) curve was also calculated to determine the optimal cutoff that maximizes sensitivity and specificity in predicting mortality. All tests of hypotheses were two-tailed and conducted at a significance level of 0.05. Statistical analyses were conducted using SAS 9.4.

## SUPPLEMENTARY MATERIALS






