# Supplementary material for: Paradoxes in thyroid carcinoma treatment: analysis of the SEER database 2010—2013
Source: Oncotarget. 2016 Nov 16;8(1):345–53. doi: 10.18632/oncotarget.13395 (PMC5352124; doi:10.18632/oncotarget.13395)
Supplement: Supplementary file 1 [file oncotarget-08-345-s001.pdf]

Paradoxes in thyroid carcinoma treatment: analysis of the SEER database 2010–2013

Supplementary Materials

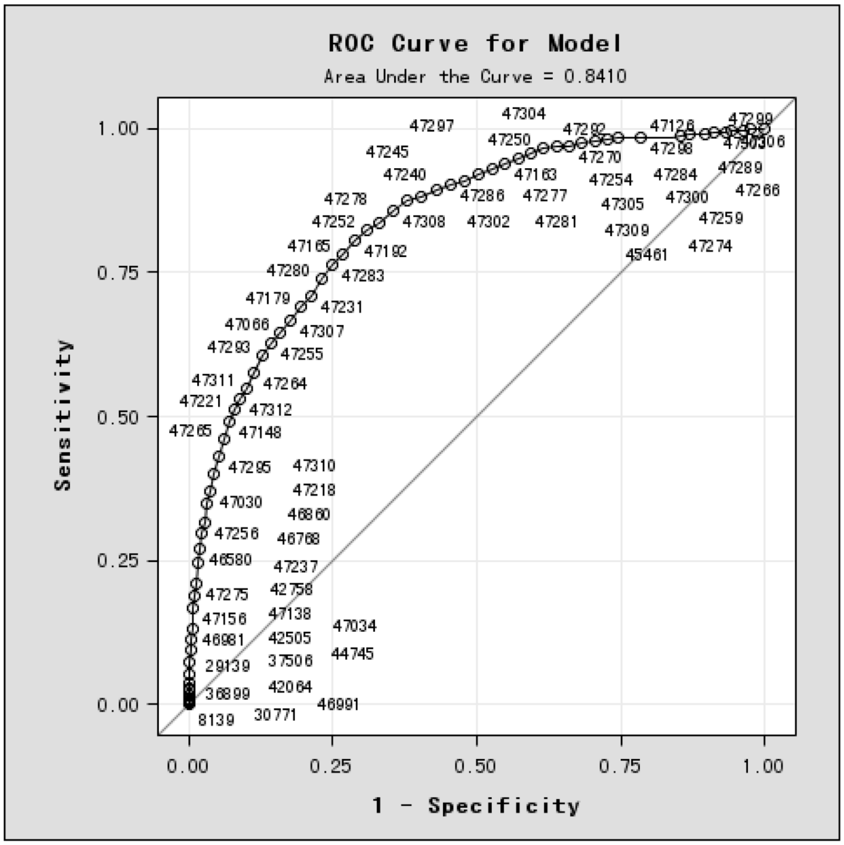

59 is the optimal cutoff for age as a predictor of thyroid carcinoma-specific mortality

ID in the graph	Age	Probability	Sensitivity	Specificity
47,278	57	0.01145	0.83700	0.66884
47,252	58	0.01264	0.82536	0.69035
47,192	59	0.01396	0.80595	0.71164
47,165	60	0.01542	0.78266	0.73154
47,283	61	0.01703	0.76455	0.75053

Supplementary Figure S1: ROC for Age (AUC = 0.8140, *P* < 0.0001): outcome is thyroid carcinoma-specific mortality (*n* = 47,312).

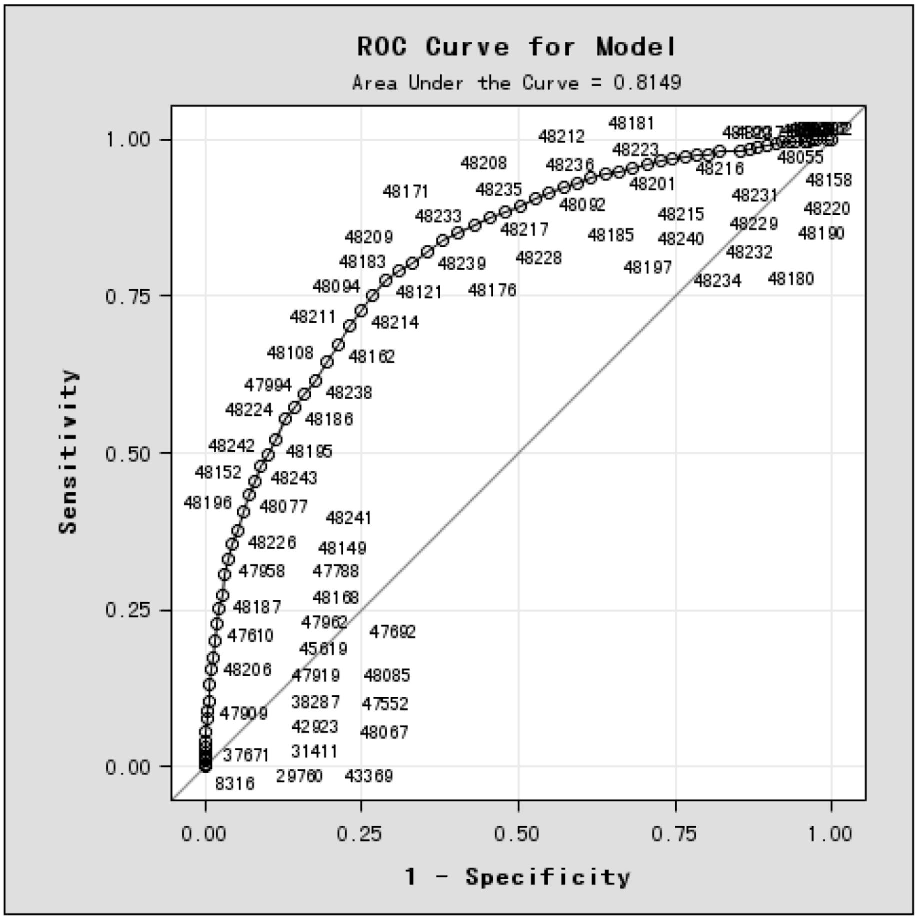

59 is the optimal cutoff for age as a predictor of all-cause mortality

ID in the graph	Age	Probability	Sensitivity	Specificity
48,209	57	0.02868	0.80399	0.66884
48,183	58	0.03125	0.79108	0.69035
48,121	59	0.03404	0.77523	0.71164
48,094	60	0.03707	0.75000	0.73154
48,214	61	0.04037	0.72711	0.75053

Supplementary Figure S2: ROC for Age (AUC = 0.8149, *P* < 0.0001): outcome is all cause mortality (*n* = 48,243).

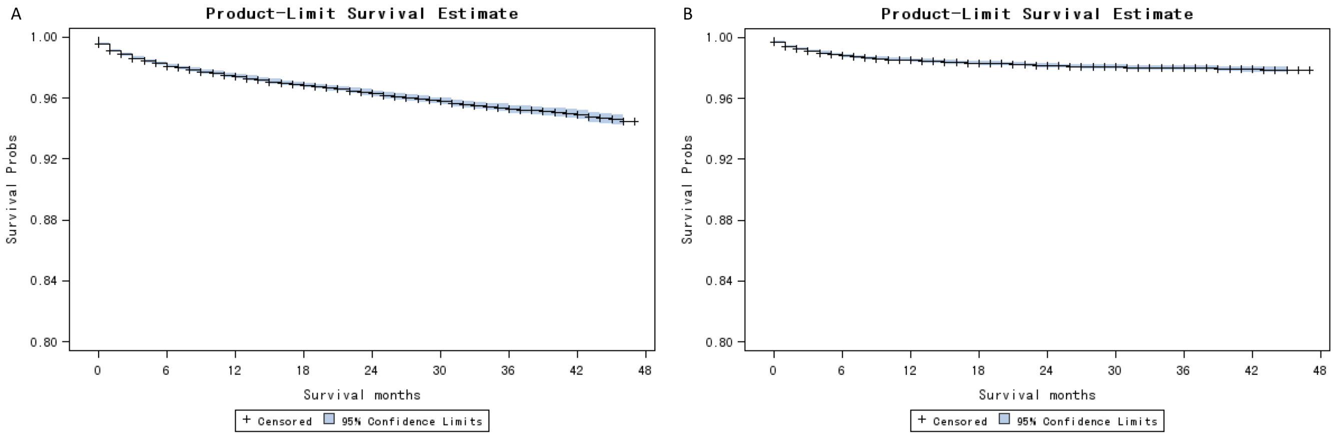

Survival rate information for thyroid carcinoma-specific mortality

Timelist (months)	Months	Survival	Number Failed	Number Left
6	6	0.9891	473	37133
12	12	0.9860	584	31259
18	18	0.9843	634	25436
24	24	0.9826	672	19722
30	30	0.9818	687	14187
36	36	0.9811	696	8909
42	41	0.9803	701	3999
48	45		703	0

Supplementary Figure S3: Kaplan Meier Curve for all-cause mortality (A) and thyroid carcinoma-specific mortality (B).

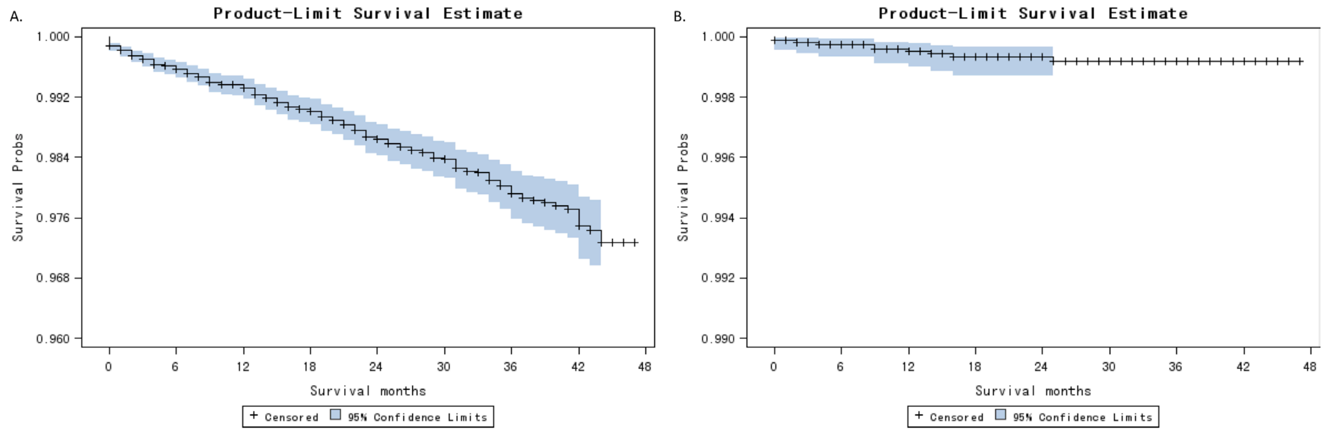

Survival rate information for thyroid carcinoma-specific mortality among patients with micro-carcinoma patients

Timelist (months)	Months	Survival	Number Failed	Number Left
6	4	0.9998	4	13796
12	12	0.9995	7	11739
18	16	0.9993	9	9529
24	16	0.9993	9	7420
30	25	0.9992	10	5355
36	25	0.9992	10	3356
42	25	0.9992	10	1487
48	25		10	0

Supplementary Figure S4: Kaplan Meier Curve for all-cause mortality (A) and thyroid carcinoma-specific mortality (B) among patients with thyroid micro-carcinoma.

Supplementary Table S1: Survival rate information for all cause mortality among patients stratified by T stage. See Supplementary_Table_S1.

Supplementary Table S2: Survival rate information for thyroid carcinoma-specific mortality among patients stratified by T stage. See Supplementary_Table_S2.

Supplementary Table S3: Survival rate information for thyroid carcinoma specific mortality among patients with thyroid micro-carcinoma stratified by surgery treatment

	No surgery (*n* = 109)				Surgery performed (*n* = 17193)			
Time list (months)	Event Months	Survival Rate	Number Failed	Number Left	Event Months	Survival Rate	Number Failed	Number Left
6	0	1.0000	0	70	4	0.9998	4	13716
12	0	1.0000	2	58	12	0.9995	7	11672
18	0	1.0000	2	49	16	0.9993	9	9475
24	0	1.0000	3	41	16	0.9993	9	7377
30	0	1.0000	3	25	25	0.9992	10	5329
36	0	1.0000	3	14	25	0.9992	10	3341
42	0	1.0000	3	6	25	0.9992	10	1481
48	0		3	0	25		10	0

Supplementary Table S4: Survival rate information for thyroid carcinoma specific mortality among patients with thyroid micro-carcinoma stratified by radiation treatment

	No radiation (*n* = 13296)				Radiation performed (*n* = 3633)			
Time list (months)	Event Months	Survival Rate	Number Failed	Number Left	Event Months	Survival Rate	Number Failed	Number Left
6	4	0.9997	4	10429	0	1.0000	0	3087
12	12	0.9995	6	8830	0	1.0000	0	2684
18	16	0.9993	7	7148	14	0.9996	1	2228
24	16	0.9993	7	5541	14	0.9996	1	1772
30	16	0.9993	7	3929	25	0.9991	2	1347
36	16	0.9993	7	2400	25	0.9991	2	898
42	16	0.9993	7	1060	25	0.9991	2	396
48	16		7	0	25		2	0
