# Supplementary material for: Paradoxes in thyroid carcinoma treatment: analysis of the SEER database 2010—2013
Source: Oncotarget. 2016 Nov 16;8(1):345–53. doi: 10.18632/oncotarget.13395 (PMC5352124; doi:10.18632/oncotarget.13395)
Supplement: Supplementary file 2 [file oncotarget-08-345-s002.docx]

**Supplementay Table 1. Survival rate information for all cause mortality among patients stratified by T stage.**

|  | T0 (n=84) | | | | T1 (n=28067) | | | | T2 (n=7727) | | | | T3 (n=9375) | | | | T4 (n=2107) | | | |
| --- | --- | --- | --- | --- | --- | --- | --- | --- | --- | --- | --- | --- | --- | --- | --- | --- | --- | --- | --- | --- |
| Time list  (months) | Event Months | Survival Rate | Number Failed | Number Left | Event Months | Survival Rate | Number Failed | Number Left | Event Months | Survival Rate | Number Failed | Number Left | Event Months | Survival Rate | Number Failed | Number Left | Event Months | Survival Rate | Number Failed | Number Left |
| 6 | 3 | 0.9512 | 4 | 68 | 6 | 0.9957 | 110 | 22450 | 6 | 0.9930 | 50 | 6223 | 6 | 0.9906 | 81 | 7574 | 6 | 0.7447 | 512 | 1320 |
| 12 | 3 | 0.9512 | 4 | 61 | 12 | 0.9927 | 174 | 18964 | 12 | 0.9898 | 69 | 5263 | 12 | 0.9848 | 122 | 6315 | 12 | 0.6766 | 627 | 1030 |
| 18 | 16 | 0.8998 | 7 | 46 | 18 | 0.9896 | 231 | 15400 | 18 | 0.9843 | 96 | 4274 | 18 | 0.9771 | 168 | 5152 | 18 | 0.6495 | 666 | 816 |
| 24 | 19 | 0.8802 | 8 | 35 | 24 | 0.9860 | 281 | 11967 | 24 | 0.9802 | 112 | 3292 | 24 | 0.9714 | 195 | 3957 | 24 | 0.6188 | 701 | 631 |
| 30 | 19 | 0.8802 | 8 | 25 | 30 | 0.9824 | 320 | 8611 | 29 | 0.9739 | 131 | 2360 | 30 | 0.9655 | 217 | 2819 | 30 | 0.6043 | 714 | 455 |
| 36 | 19 | 0.8802 | 8 | 22 | 36 | 0.9783 | 350 | 5382 | 36 | 0.9691 | 141 | 1455 | 36 | 0.9599 | 231 | 1774 | 34 | 0.5943 | 721 | 307 |
| 42 | 19 | 0.8802 | 8 | 11 | 42 | 0.9752 | 362 | 2396 | 42 | 0.9663 | 144 | 646 | 41 | 0.9558 | 237 | 835 | 41 | 0.5843 | 725 | 122 |
| 48 | 46 |  | 9 | 0 | 44 |  | 368 | 0 | 42 |  | 144 | 0 | 46 |  | 241 | 0 | 43 |  | 727 | 0 |
