# Supplementary material for: Paradoxes in thyroid carcinoma treatment: analysis of the SEER database 2010—2013
Source: Oncotarget. 2016 Nov 16;8(1):345–53. doi: 10.18632/oncotarget.13395 (PMC5352124; doi:10.18632/oncotarget.13395)
Supplement: Supplementary file 3 [file oncotarget-08-345-s003.docx]

**Supplementay Table 2. Survival rate information for thyroid carcinoma-specific mortality among patients stratified by T stage.**

|  | T0 (n=78) | | | | T1 (n=27657) | | | | T2 (n=7607) | | | | T3 (n=9217) | | | | T4 (n=1939) | | | |
| --- | --- | --- | --- | --- | --- | --- | --- | --- | --- | --- | --- | --- | --- | --- | --- | --- | --- | --- | --- | --- |
| Time list  (months) | Event Months | Survival Rate | Number Failed | Number Left | Event Months | Survival Rate | Number Failed | Number Left | Event Months | Survival Rate | Number Failed | Number Left | Event Months | Survival Rate | Number Failed | Number Left | Event Months | Survival Rate | Number Failed | Number Left |
| 6 | 1 | 0.9742 | 2 | 64 | 6 | 0.9996 | 10 | 22207 | 5 | 0.9977 | 16 | 6142 | 6 | 0.9956 | 37 | 7462 | 6 | 0.7447 | 408 | 1258 |
| 12 | 1 | 0.9742 | 2 | 57 | 12 | 0.9992 | 18 | 18777 | 12 | 0.9970 | 20 | 5197 | 12 | 0.9938 | 50 | 631 | 12 | 0.6766 | 494 | 997 |
| 18 | 1 | 0.9742 | 2 | 45 | 18 | 0.9990 | 22 | 15266 | 17 | 0.9960 | 25 | 4230 | 18 | 0.9918 | 62 | 5102 | 18 | 0.6495 | 523 | 793 |
| 24 | 19 | 0.9525 | 3 | 34 | 20 | 0.9989 | 23 | 11882 | 21 | 0.9957 | 26 | 3264 | 24 | 0.9896 | 72 | 3924 | 24 | 0.6188 | 548 | 618 |
| 30 | 19 | 0.9525 | 3 | 24 | 30 | 0.9987 | 25 | 8563 | 27 | 0.9954 | 27 | 2349 | 30 | 0.9888 | 75 | 2805 | 30 | 0.6043 | 557 | 446 |
| 36 | 19 | 0.9525 | 3 | 21 | 30 | 0.9987 | 25 | 5364 | 33 | 0.9949 | 28 | 1455 | 36 | 0.9864 | 81 | 1768 | 34 | 0.5943 | 559 | 303 |
| 42 | 19 | 0.9525 | 3 | 10 | 30 | 0.9987 | 25 | 2396 | 39 | 0.9940 | 29 | 646 | 41 | 0.9848 | 83 | 833 | 41 | 0.5843 | 561 | 120 |
| 48 | 19 |  | 3 | 0 | 30 |  | 25 | 0 | 39 |  | 29 | 0 | 45 |  | 85 | 0 | 43 |  | 561 | 0 |
